# A Low-cost Electromagnetic Docking Guidance System for Micro Autonomous Underwater Vehicles

**DOI:** 10.3390/s19030682

**Published:** 2019-02-07

**Authors:** Shilin Peng, Jingbiao Liu, Junhao Wu, Chong Li, Benkun Liu, Wenyu Cai, Haibin Yu

**Affiliations:** 1 College of Electronics and Information, Hangzhou Dianzi University, Hangzhou 310018, China; psl@hdu.edu.cn (S.P.); ab@hdu.edu.cn (J.L.); wujunhow@163.com (J.W.); lichong1426@163.com (C.L.); benkun_liu@163.com (B.L.); caiwy@hdu.edu.cn (W.C.); 2 Zhejiang Provincial Key Lab of Equipment Electronics, Hangzhou 310018, China

**Keywords:** autonomous underwater vehicles, electromagnetic docking, three-axial search coil, magnetometer, low frequency

## Abstract

As important observational platforms for the Smart Ocean concept, autonomous underwater vehicles (AUVs) that perform long-term observation in fleets are beneficial because they provide large-scale sampling data with a sufficient spatiotemporal resolution. Therefore, a large number of low-cost micro AUVs with docking capability for power recharge and data transmission are essential. This study designed a low-cost electromagnetic docking guidance (EMDG) system for micro AUVs. The EMDG system is composed of a transmitter coil located on the dock and a three-axial search coil magnetometer acting as a receiver. The search coil magnetometer was optimized for small sizes while maintaining sufficient sensitivity. The signal conditioning and processing subsystem was designed to calculate the deflection angle (*β*) for docking guidance. Underwater docking tests showed that the system can detect the electromagnetic signal and successfully guide AUV docking. The AUV can still perform docking in extreme positions, which cannot be realized through normal optical or acoustic guidance. This study is the first to focus on the EM guidance system for low-cost micro AUVs. The search coil sensor in the AUV is inexpensive and compact so that the system can be equipped on a wide range of AUVs.

## 1. Introduction

The Smart Ocean concept tries to comprehensively enhance the capabilities of the ocean using big data technology, and it is inseparable from the support of technology in the areas of marine sensing and control. Obviously ocean observing systems for data collection are the key enabler of the “Smart Ocean”. Autonomous underwater vehicles (AUVs) that can conduct spatiotemporal surveys and collect data, which are impossible via other methods [1], are useful platforms for ocean observation. Docking an AUV for power recharge and data transmission is beneficial for applications that require long-term observation. This scheme can reduce the need for frequent launch and recovery and enable AUVs to permanently reside in a specific area to be ready for underwater operations [2]. Moreover, if the docking station is connected to a cabled ocean observatory, then the AUVs can be considered additional mobile nodes for 3D ocean observation [3].

As important observational platforms for Smart Ocean, many AUVs that conduct long-term observation are needed so that large-scale sampling data with a sufficient spatiotemporal resolution can be acquired. Therefore, low-cost micro AUVs with docking capability for power recharge and data transmission are crucial. 

In accordance with the performance of navigation devices, the docking process can be divided into two sequences, namely, a long-distance homing stage in which the AUV performs homing from more than 100 to 200 m away to the close proximity of a docking station and a proximal docking stage in which the AUV maneuvers into the docking station [4]. The docking stage, which is also called the terminal guidance stage, is important but difficult because the AUV needs to steer into the docking station precisely under certain guidance. Several methodologies address docking guidance, and these include optical or visual guidance [2,4,5,6,7,8,9], acoustic guidance [10,11,12,13,14,15], electromagnetic (EM) guidance [16,17], electric sense [18], and different combinations of these techniques [19,20,21,22,23].

Among these docking guidance methodologies, acoustic systems are proven effective for position finding at distances less than 1 m [16] and for long-distance homing. However, in the proximal docking stage where precise spatial measurements with a fairly rapid temporal resolution are required, an acoustic guidance system requires high update rates and extensive signal conditioning, which is a challenge for acoustic systems [16]. In addition, an acoustic system demonstrates increased complexity when it operates near the surface, bottom, or other acoustically reflective boundaries [16,17]. Optical or visual guidance systems provide good response in non-turbid, non-fouling environments but may be limited in turbid and bio-fouling-prone environments [16]. The EM guidance system is immune to several of the factors that affect other systems, such as water turbidity, biofouling, mists or clusters of bubbles, and acoustically reflective boundaries [16]. Therefore, EM guidance is an alternative technology that demonstrates robust operation under complex underwater conditions.

In their study of EM guidance, Feezor et al. [16] designed an EM homing system with an effective range of 25–30 m. The EM homing system consists of a transmitter located on the dock and a receiver mounted on the AUV. The transmitter generates two magnetic dipole fields from two coils. A horizontally disposed coil generates a 1 kHz field for distinguishing the front of the dock from the rear, and another vertically disposed coil emits a 2 kHz field for directional control. The receiver coil in the nose of the AUV consists of three orthogonal coils with a coil diameter of 90 mm each. Vandavasi et al. [17] presented an EM homing guidance system composed of an underwater dock with an EM dipole coil and a twin-thruster AUV equipped with a differential magnetometer system. By undergoing a 360° rotation and measuring the magnetic field gradient, the AUV can calculate the bearing angle, which is used for docking guidance. This guidance system has an effective range of 7 m. 

However, little attention has been paid to the EM guidance system for low-cost micro AUVs; this system requires that the receiver on the AUV should be inexpensive and small while maintaining sufficient sensitivity. In this manner, the EM guidance system can be adopted in a wide range of AUVs, especially low-cost micro ones. 

The current study designed a low-cost EM docking guidance (EMDG) system for micro AUVs. The EMDG system is composed of a transmitter coil located on the dock and a three-axial search coil magnetometer acting as a receiver. We optimized the search coil magnetometer for small size and high sensitivity and designed a signal conditioning and processing subsystem to extract the docking guidance signal.

This work contributes to the field of AUV docking, which provides long-term mobile and autonomous ocean sampling for Smart Ocean [10,24]. The importance and originality of this study are demonstrated by the fact that this study is the first to focus on the EM guidance system for low-cost micro AUVs. With a large number of low-cost micro AUVs with EM docking capability, large-scale sampling data with sufficient space resolution can be achieved. 

The remainder of this paper is organized as follows: Section 2 presents the EM docking guidance principles and the EMDG system. The design and optimization of the search coil magnetometer for small size and high sensitivity are descried in detail in Section 3. Section 4 addresses the design of the signal conditioning and processing subsystem of the EMDG system. Experimental results, including those of the search coil magnetometer and underwater docking tests, are presented in Section 5. Section 6 presents the conclusions. 

## 2. EM Docking Guidance Principles and the EMDG System

**Figure 1:**
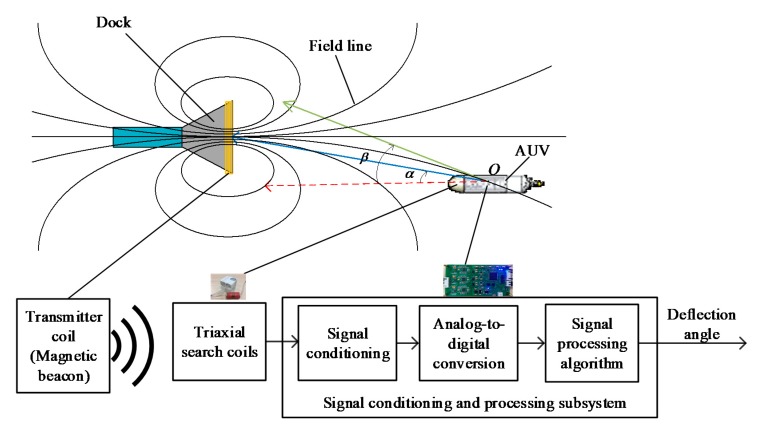
Schematic of AUV docking via electromagnetic (EM) guidance and structure of electromagnetic docking guidance (EMDG) system.

The basic principle of the proximal AUV EM docking guidance is shown in Figure 1. A set of transmitter coil encircling the dock entrance is acting as a magnetic beacon. A magnetic field can be generated after the electrification of magnetic coil. For an air-core coil, the magnetic moment generated by the coil can be defined as the product of the exciting current *I*, the number of turns *N*, and the area *S*, that is, the magnetic moment *M* = *NSI*. The magnetic moment vector ***M*** is a vector with magnitude *M* and a direction coincided with the axis of the coil. Given that the focused area is near the field, that is, the distance is shorter than the wavelength of the electromagnetic wave, the magnetic field ***B*** at distances larger than the coil’s largest dimension can be expressed as a magnetic dipole field:

(1) $$B=\frac{\mu_{{}_{0}}}{4\pi r^{5}}\left[3(M\cdot r)r-Mr^{2}\right]$$

where *μ*_0_ is the permeability of vacuum, which is 4π × 10^−7^ (H/m), and ***r*** is position vector from the center of the coil to the measurement point.

According to the characteristics of the magnetic dipole model, all the magnetic field lines will pass through the magnetic coil, that is, the dock entrance. Therefore, the EM signal can be used as the guiding signal of AUV docking. The bearing angle between AUV and magnetic beacon is *α*, and the angle between the axis of AUV and the tangent direction of magnetic field is defined as the deflection angle *β*. The current bearing angle *α* can be obtained by optical and acoustic guidance, which are both guided by taking the bearing as the datum. The EM guidance mode adopted by this paper takes the deflection angle *β* as the datum. That is, the AUV regards the deflection angle *β* of the current position as the yaw angle to make the course of the AUV consistent with the current magnetic field direction, which is the controlling target, to control the AUV entrance to the dock along the magnetic field line and complete the docking process.

There are generally two types of docks [16]. One is a conical dock which has a cone shape entry. The other is an omnidirectional dock that can be approached from any direction. Given that the conical entry system provides a simple dock design, the present study has adopted a conical dock. Considering that the proximal docking stage is more critical than the long-distance homing stage, the present study focuses on the EM docking guidance in the proximal docking stage. In the beginning of this stage, we assumed that the AUV has arrived at the close proximity of a docking station by the homing guidance of an acoustic system, which is approximately 10–20 m ahead of the dock entrance. In addition, the AUV knows the heading and depth of the dock beforehand through the acoustic communication between the dock and the AUV. Therefore, there is no need for the EM guidance system to distinguish the front of the dock from the rear. 

The structure of the EMDG system is also shown in Figure 1. It consists of a magnetic beacon, triaxial search coils, and a signal conditioning and processing subsystem, which includes a signal conditioning circuit, an analog-to-digital conversion circuit, and a guidance signal processing algorithm.

The high frequency of electromagnetic signal leads to strong attenuation by sea water, and the low signal frequency can affect the real-time performance of guidance. Considering the trade-off between effective distance and real-time performance, we chose 1–2 kHz as the frequency of the guidance signal. In the process of proximal docking, the intensity of the electromagnetic signal is approximately (10^−5^–10^−10^) T. Currently, mature and commercialized triaxial magnetic sensors include magnetoresistive series and fluxgate sensors, which are traditionally used for static magnetic measurements. For example, the resolution of HMC series magnetoresistive sensor of Honeywell Company is less than 10^−^^9^ T [25]. The fluxgate sensor can measure the level at 10^−11^ T [26]; however, this sensor has reached the measuring limit of high-end fluxgates and the cost is high. The search coil sensor is also called an induction coil sensor, with a wide measurement range of approximately (10^−12^–10^3^) T and wide frequency bandwidth [27]. This type of magnetic sensor is practically the only one that can be made directly by users themselves by winding the coil [27], and has been demonstrated in applications of magnetic positioning on land [28,29,30,31,32]. Therefore, we chose the search coil sensor as the receiver sensor of the EMDG system. 

In the literature [16], the search coil sensor of the EM guidance system consists of three orthogonal coils with each coil having a 90 mm diameter. The sensor was mounted in a mid-size Odyssey IIb AUV with a 580 mm diameter. Because the sensor has a large volume, it is not suitable for some low-cost portable micro AUVs, which have a typical hull diameter of (110–150) mm. Therefore, we optimized the search coil sensor for small size and high sensitivity and designed a signal conditioning and processing subsystem to extract the docking guidance signal. These will be presented in detail in the following sections.

## 3. Design and Optimization of Search Coil Sensor

### 3.1. Operational Principles

According to Faraday’s law, the closing coil can generate induced current in a magnetic field with variable magnetic flux. If the magnetic beacon emits an electromagnetic field $B=B_{in}\cos(2\pi ft)$, then the voltage generated by the coil *V* is:

(2) $$V=-nS_{air}\frac{dB}{dt}=2\pi fnS_{air}B_{in}\sin\left(2\pi ft\right),$$

where *n* is the number of coils, and *S*_air_ is the effective area of magnetic flux. The output voltage *V* is proportional to the amount of change in magnetic induction intensity *B_in_* over time. The sensitivity of the sensor is defined as:

(3) $$S=\frac{V}{B}=2\pi fnS_{eff}.$$

According to Equation (3), when the frequency is fixed, the number of coils and effective area of magnetic flux can be increased to improve the sensitivity of the coil sensor. However, due to the limited application space, the number of coils and the effective area of magnetic flux cannot be infinitely increased. According to the size of the typical portable micro AUVs, which has a typical hull diameter of (110–150) mm, this paper limited the size of the uniaxial sensor to length of *Lc* = 50 mm and diameter of *D* = 25 mm. Through an analysis of the other variable factors of the sensor, the sensitivity of the sensor was optimized to improve the recognition distance of the AUV electromagnetic guidance system.

### 3.2. Structural Design of Magnetic Core

**Figure 2:**
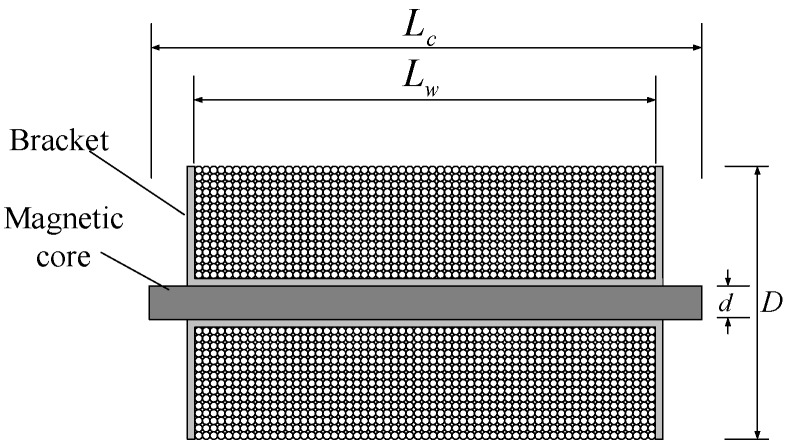
Structure of magnetic core coil sensor.

The volume of the air–core coil sensor limits the improvement of sensitivity. A soft magnetic core with high magnetic permeability can be used as the magnetic core to improve the sensitivity of the sensor, thus breaking the volume limit to solve the aforementioned problem [27]. The structure of magnetic core coil sensor is shown in Figure 2.

The soft magnetic field to be magnetized is placed on the magnetic field strength H, and the magnetization intensity is M=χH, where χ is the magnetic susceptibility. Then, the magnetic induction intensity B of the coil sensor [33] is:

(4) $$B=\mu_{0}\left(H+M\right)=\mu_{0}\left(1+\chi \right)H=\mu_{0}\mu_{r}H.$$

The magnetic induction of air-core coil sensor is $B_{0}=\mu_{0}H$. The high relative magnetic permeability $\mu_{r}$ of the soft magnetic field results in a part of extra high magnetic induction $B_{core}=\mu_{0}\chi H$ being produced to improve the sensitivity of the sensor.

However, the soft magnetic field in the magnetized state will generate a demagnetization field with the opposite direction to the original magnetic field, producing a demagnetization effect and causing the decrease in *B_core_*. The demagnetization effect depends on the demagnetization factor *N*, which is related to the geometry of the core only [27]. The ratio between *B_core_* and the external magnetic field outside the sensor (*B*_ext_) is the apparent permeability $\mu_{app}$ [34], and its effect must be considered when designing the sensor. 

**Figure 3:**
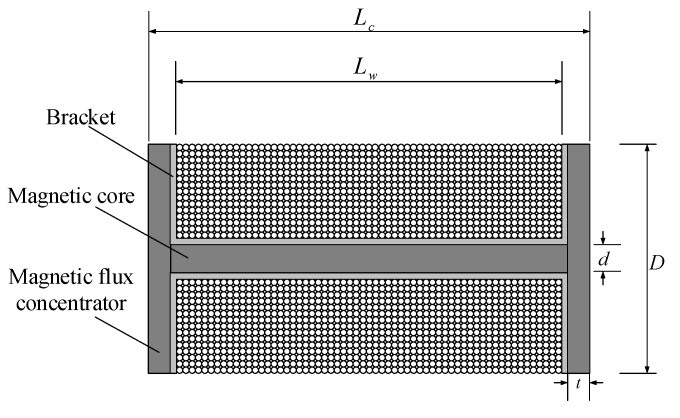
Structure of the improved magnetic core coil sensor.

**Figure 4:**
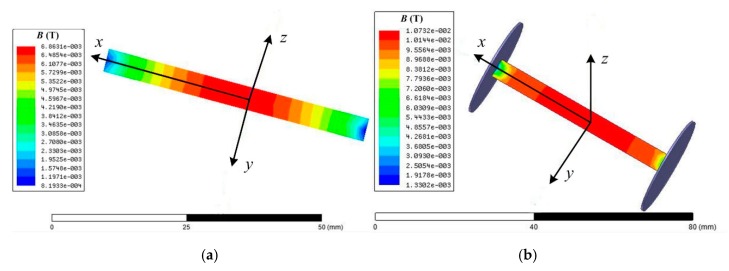
Magnetic induction distribution of magnetic core in a constant magnetic field. (**a**) Without magnetic flux concentrator. (**b**) With magnetic flux concentrator.

In a small magnetic core coil sensor, the core geometry can be changed to slow down the drop of apparent permeability, concentrate the magnetic core with additional magnetic flux, and enhance the magnetic induction intensity. As shown in Figure 3, two thin and wide flat disk magnetic flux concentrators were placed at the two ends of the core. The finite element analysis software Maxwell was used to analyze the distribution of the magnetic induction intensity of the core in a constant magnetic field. Figure 4a,b show the result of the magnetic core without and with magnetic flux concentrator, respectively. The comparison demonstrates that the magnetic flux concentrators can improve the distribution unevenness of apparent permeability caused by the demagnetization effect. This method can improve the sensitivity of the sensor and reduce the length of the core, which is conducive to the miniaturization of the sensor. Based on Equation (3), the sensitivity of the magnetic core search coil sensor with magnetic flux concentrator can be rewritten as:

(5) $$S=2\pi fnS_{c}\mu_{app},$$

where effective area of the coil magnetic flux is the cross-sectional area of the core *S_c_*. When the soft magnetic relative permeability is $\mu_{r}$ ≫ 1, the soft magnetic apparent permeability $\mu_{app}$ is [34,35]:

(6) $$\mu_{app}=\frac{\mu_{r}}{1+\mu_{r}Nd^{2}/D^{2}},$$

(7) $$N=\frac{1}{m^{2}-1}\left[\frac{m}{\sqrt{m^{2}-1}}\ln\left(m+\sqrt{m^{2}-1}\right)-1\right],$$

where *m* = *L*_c_/*D*, *N* is the demagnetization factor.

### 3.3. Parameter Optimization

**Figure 5:**
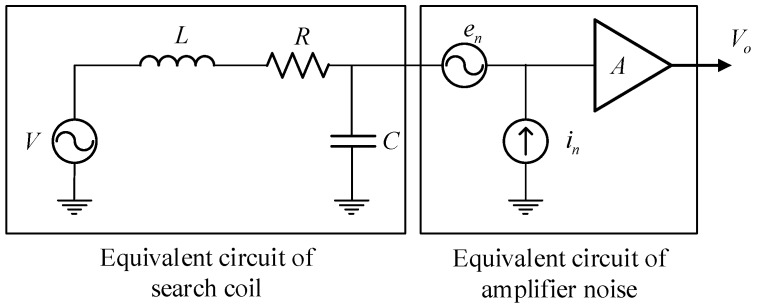
Equivalent circuit of search coil sensor.

The equivalent circuit of search coil sensor is shown in Figure 5, which is composed of induced voltage source *V*, inductance *L*, resistance *R*, and wire-to-wire capacitance *C* [34]. The voltage output is weak and thus needs to be amplified by the amplifier. Given that the sensitivity of the sensor is affected by noise, the magnetic noise (magnetometer sensitivity threshold) *B_st_* is defined as the ratio of the total noise spectral density to the induced voltage [34], given by:

(8) $$B_{st}=\frac{\sqrt{4kTR+e_{n}{}^{2}+{\left(Ri_{n}\right)}^{2}}}{2\pi fnS_{c}\mu_{app}},$$

where the total noise spectral density $e_{all}=\sqrt{4kTR+e_{n}{}^{2}+{\left(Ri_{n}\right)}^{2}}$ is composed of coil resistance thermal noise $\sqrt{4kTR}$, preamplifier input voltage noise *e_n_*, and input current noise *i_n_*. The number of coils n is determined by the wire diameter (with insulating layer) and the size of the bracket *D*, given by:

(9) $$n=\frac{L_{w}(D-d)}{2d_{w}{}^{2}}.$$

The sectional area of magnetic core *S_c_* is:

(10) $$S_{c}=\frac{{\pi d}^{2}}{4}.$$

Coil resistance *R* is computed as follows:

(11) $$R=2n\rho \frac{D+d}{d_{w}{}^{2}}.$$

With the use of Equations (5)–(11), the sensitivity at this time can be obtained as:

(12) $$S=\frac{\pi^{2}fL_{w}(D-d)d^{2}\mu_{app}}{4d_{w}{}^{2}}.$$

According to Equations (8)–(12), the sensitivity of the sensor and magnetic noise is related to various parameters. However, the parameters that can be optimized include the winding wire diameter *d_w_* and the core diameter *d* under limited conditions.

**Figure 6:**
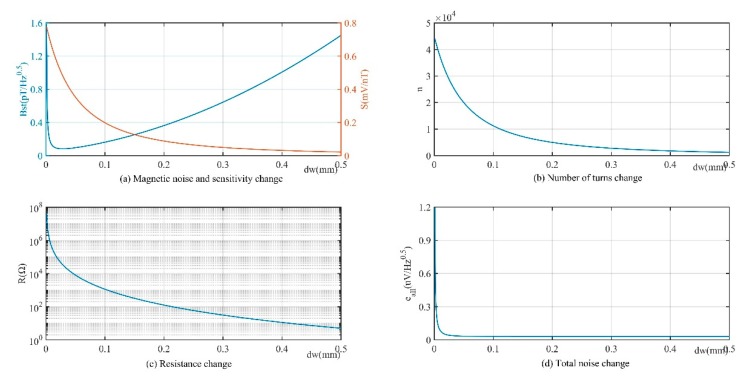
Relation curve between magnetic noise, sensitivity, number of coils, resistance, total noise spectral density, and wire diameter *d_w_* (Fixed parameters Lc=5 cm, Lw=4.5 cm, d=5 mm, $\rho =1.678\times 10^{-8}\;\Omega \cdot m$, $e_{n}=32\;\mathrm{nV}/\sqrt{\mathrm{Hz}}$, $i_{n}=20\;\mathrm{fA}/\sqrt{\mathrm{Hz}}$, $\mu_{r}=2\times 10^{4}$, D=2.5 cm, and f=1 kHz ).

Figure 6 is the relation curve between sensor sensitivity and magnetic noise and other parameters when the line diameter *d_w_* is in the range of 0.01–0.5 mm. Figure 6a shows that the sensitivity is high when the wire diameter is fine, which can also be suggested from the inverse relation between sensitivity *S* and *d*_*w*_^2^ in Equation (12). The magnetic noise increased as the wire diameter increased from 0.02 mm. Although the increase in wire diameter reduced the resistance, which decreased the resistance thermal and current noise, the changes in the two, as shown in Figure 6d, account for only 0.0976% of the voltage noise, which can be negligible. Thus, according to Equations (8)–(10), magnetic noise *B_st_* is proportional to *d*_*w*_^2^. When the wire diameter *d_w_* decreased from 0.02 mm as shown in Figure 6c (the coordinate is the logarithmic scale), the coil resistance increased rapidly, and the total noise was dominated by resistance thermal and current noise, which led to the rise of magnetic noise.

**Figure 7:**
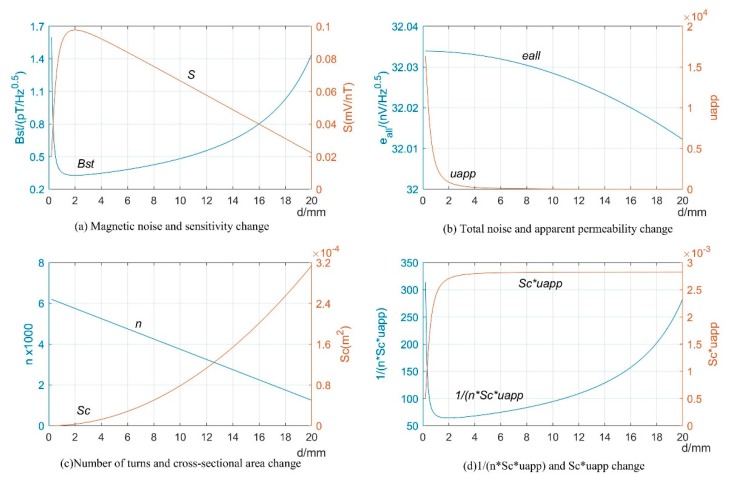
Relation curve between magnetic noise, sensitivity, total noise spectral density, apparent permeability, cross-sectional area $1/nS_{c}\mu_{app}$, $S_{c}\mu_{app}$, and core diameter *d*. The fixed parameter is *d_w_* = 0.2 mm, and other parameters are consistent with Figure 6.

Figure 7 is the relation curve between sensor sensitivity and magnetic noise and other parameters with the change of magnetic core diameter *d* when it is in the range of 0.2–20 mm. Figure 7b shows that the total noise spectral density changed slightly at this time, which can be ignored. Figure 7a demonstrates that the sensitivity changed in the opposite trend with the magnetic noise, which showed an extreme point at 2 mm. Figure 7d shows that $1/nS_{c}\mu_{app}$ was consistent with the trend of magnetic noise curve. When *d* > 2 mm, the apparent permeability was $\mu_{app}\approx D^{2}/Nd^{2}$; then:

(13) $$S\approx \frac{\pi^{2}fL_{w}(D-d)D^{2}}{4Nd_{w}{}^{2}}$$

This finding suggests that when *d* > 2 mm, the sensitivity *S* is approximately linear with *D*. However, when the diameter of the magnetic core decreased from 2 mm, the correlation curve observation of $S_{c}$ and $\mu_{app}$ showed that, despite the increase in magnetic permeability, the sectional area of the magnetic core reduced to a large extent, which resulted in a decrease in sensitivity and an increase in magnetic noise.

**Figure 8:**
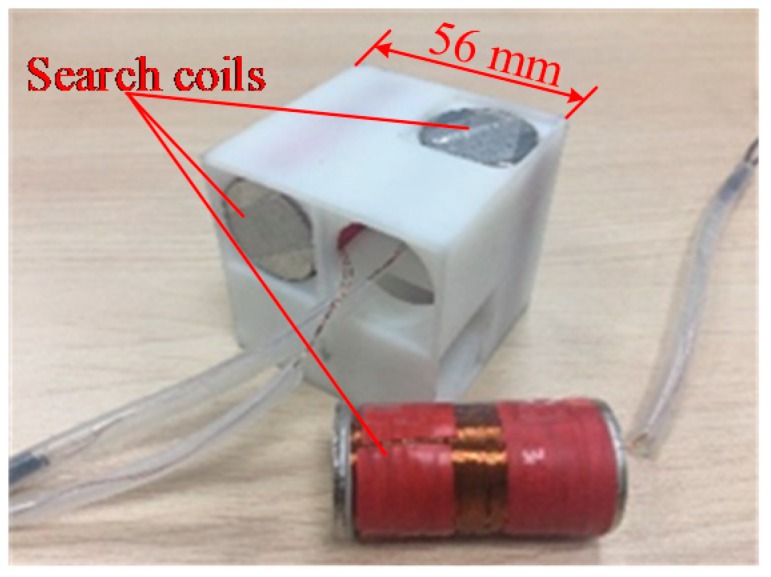
Triaxial search coil sensor.

From the preceding analysis of the variable parameters of the search coil sensor, and combining the constraint parameters and considering the complexity of the machining process, the wire diameter *d_w_* = 0.2 mm and core diameter *d* = 5 mm were selected, and 1J50 soft magnetic alloy was chosen as the core material. A cubic triaxial crossing structure was assembled as shown in Figure 8 to achieve the triaxial orthogonal effect while maintaining a small space. The size of the triaxial search coil sensor was 56 mm × 56 mm × 56 mm, and the space utilization ratio was approximately 75%. This structure can ensure that the triaxial sensor coils do not shift with the vibration of external mechanics and lose their orthogonal characteristics.

## 4. Design of the Signal Conditioning and Processing Subsystem

**Figure 9:**
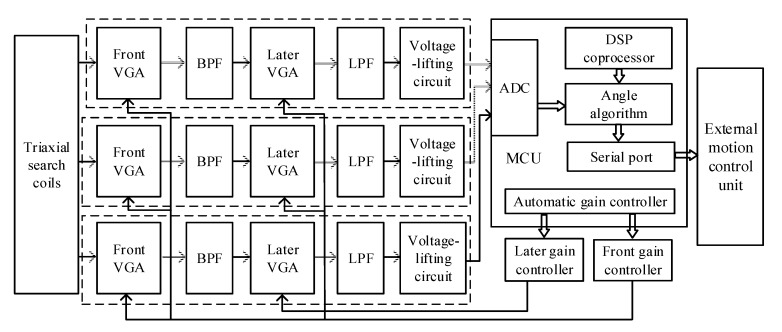
Diagram of the signal conditioning and processing subsystem (BPF: Band-pass filter; VGA: Variable gain controller; LPF: Low-pass filter).

The low-frequency electromagnetic waves propagate in a vacuum and attenuate by the cube of the distance. The effect of seawater on its attenuation should also be considered in the seawater. When electromagnetic wave frequency *f* = 1 kHz, the skin depth is $\delta =1/\sqrt{\pi f\mu_{0}\sigma }=7.9576$, where the conductivity of the seawater σ=4Ω/m [33]. This means that the intensity of the electromagnetic wave would be attenuated by *e*^−1^ = 0.3578 times for each skin depth passing the magnetic field. When the AUV proximal docking distance is larger than 5 m, the amplitude of the voltage signal outputted by the coil sensor varies greatly. When the distance is far from the dock entrance, the electromagnetic signal received by the sensor is weak; when the distance is near the dock entrance, a strong signal will be received. The test results showed that the peak voltage signal of approximately 4.4 V can be sensed at the center of the magnetic beacon. Therefore, the rear stage circuit should be equipped with automatic variable gain amplification. As shown in Figure 9, the circuit part of the system mainly consists of three synchronous analog signal-processing circuits, a second-level automatic gain amplifier, an STM32F407 microcontroller (including an ADC conversion module and a DSP coprocessor), and a power module (not shown in the diagram).

**Figure 10:**
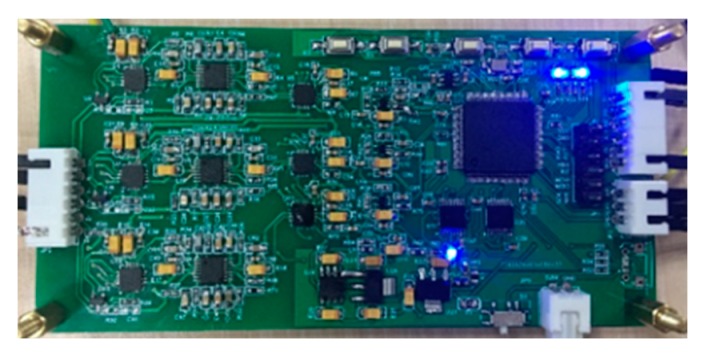
Circuit board of the signal conditioning and processing subsystem.

The analog signal conditioning circuit was powered by a ±2.5 V dual supply to provide a stimulation site with low ripples and reduce the system noise. The signal was primarily amplified by the front variable gain instrument amplifier AD8231. ADG633 was the logic level conversion chip to control the amplification gain, which provided gain selection from 1 to 128. After primary amplification, the signal filtered high- and low-frequency noise through a bandpass filter with a gain of 1, and the later variable gain instrument amplifier amplified and filtered the signal again. Given the unipolarity of ADC, the bipolar signals need to be transformed to unipolar by lifting the voltage to meet the sampling input voltage range. The circuit board of the electromagnetic guidance system is shown in Figure 10. Through the bandpass filter and lowpass filter in the signal conditioning circuit, the frequency bandwidth was restricted to (400–3000) Hz, which suppresses the noise and provides a good signal-to-noise ratio (SNR) when the frequency of signals is 1 kHz.

**Figure 11:**
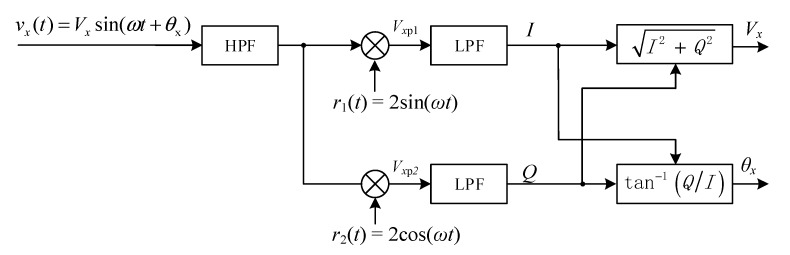
Functional block diagram of algorithm for uniaxial digital orthogonal phase-locked amplifier. It is used to calculate the voltage amplitude and phase of the *X*-axis. The calculate method in *Y*-axis and *Z*-axis are similar).

When the induced voltage signals are collected from the system, the guidance deflection angle *β* between the electromagnetic signal and AUV cannot be obtained directly. This issue needs to be solved by using the angle algorithm method. With the assistance of the DSP coprocessor, this system used the digital orthogonal phase-locked amplifier algorithm [36,37] as shown in Figure 11 to calculate the amplitudes (*V_x_*, *V_y_*, and *V_z_*) and phase (*θ_x_*, *θ_y_*, and *θ_z_*). 

Figure 11 shows the diagram of algorithm for uniaxial digital orthogonal phase-locked amplifier. We define three of the orthogonal sensor coils as X-coil, Y-coil, and Z-coil, and define the axes of them as *X*-axis, *Y*-axis, and *Z*-axis, respectively. The output signal of the X-coil is:

(14) $$v_{x}(t)=V_{x}\sin(\omega t+\theta_{x}),$$

where *V_x_* is the amplitude, *ω* is the angular frequency, *t* is time, and *θ_x_* is the phase. The signal *v_x_*(*t*) is prefiltered by a high-pass filter (HPF) with a cutoff frequency of 800 Hz, which is used for suppressing low frequency noise. The reference signals are tuned to the magnetic beacon’s frequency, given by *r*_1_(*t*) = 2sin(*ωt*) and *r*_2_(*t*) = 2cos(*ωt*). By multiplying prefiltered *v_x_*(*t*) with the reference signals, we have:

(15) $$V_{xp1}=V_{x}\sin(\omega t+\theta_{x})\times 2\sin\omega t=V_{x}\cos\theta_{x}-V_{x}\cos(2\omega t+\theta_{x}),$$

(16) $$V_{xp2}=V_{x}\sin(\omega t+\theta_{x})\times 2\cos\omega t=V_{x}\sin\theta_{x}+V_{x}\sin(2\omega t+\theta_{x}).$$

By applying low-pass filters (LPF) with cutoff frequency of 500 Hz, we get the direct current signals, that is:

(17) $$I=V_{x}\cos\theta_{x},$$

(18) $$Q=V_{x}\sin\theta_{x}.$$

Then the amplitude of the magnetic signal *V_x_* and the phase *θ_x_* are computed as follows:

(19) $$\sqrt{I^{2}+Q^{2}}=V_{x}\sqrt{\left(\sin^{2}(\theta_{x})+\cos^{2}(\theta_{x})\right)}=V_{x},$$

(20) $$\tan^{-1}\frac{Q}{I}=\tan^{-1}\frac{A\sin(\theta_{x})}{A\cos(\theta_{x})}=\theta_{x}.$$

**Figure 12:**
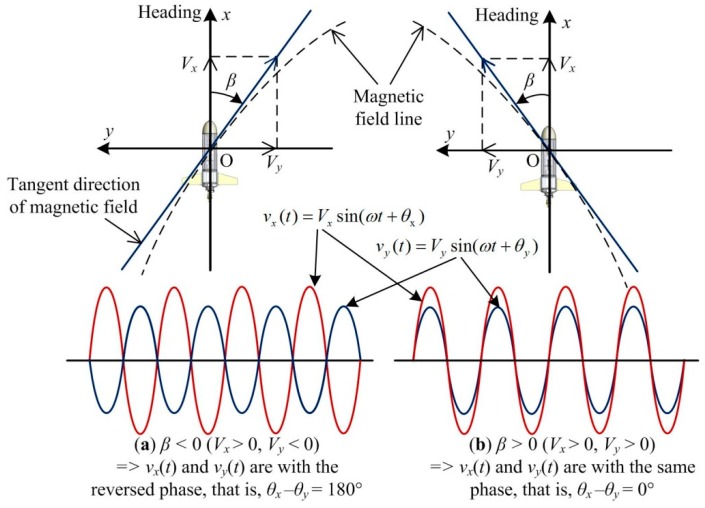
Scenarios when deflection angle in horizontal plane *β* < 0 and *β* > 0. (**a**) *β* < 0; (**b**) *β* > 0.

The signal amplitude and phase in *Y*-axis and *Z*-axis can be calculated in the same way. Then the deflection angle *β* in the horizontal plane can be calculated. As shown in Figure 12, the *X*-axis and *Y*-axis of the sensor coil are placed in the horizontal plane, and *X*-axis is aligned with the AUV heading. The absolute value of *β* can be obtained by:

(21) $$\left|\beta \right|=\arctan\left(\frac{V_{y}}{V_{x}}\right),$$

and the sign of *β* is determined by the phase difference between *θ_x_* and *θ_y_*. 

Figure 12a shows the scenario when *β* < 0. The magnetic signal sensed by the receiver coil would be *V_x_* > 0 and *V_y_* < 0. This means the signal *v_x_*(*t*) and *v_y_*(*t*) are with the reversed phase, that is, |*θ_x_* − *θ_y_*|= 180°. Similarly, Figure 12b shows the scenario when *β* > 0. The magnetic signal sensed by the receiver coil would be *V_x_* > 0 and *V_y_* > 0. This means the signal *v_x_*(*t*) and *v_y_*(*t*) are with the same phase, that is, *θ_x_* − *θ_y_* = 0°. Therefore, in the ideal condition, the phase difference |*θ_x_* − *θ_y_*| has only two values, 0° or 180°. However, due to the measurement and signal processing error, the phase difference will deviate from 0° or 180°. So the calculation of the deflection angle *β* is revised as:

(22) $$\beta =\left\{ \begin{array}{l} \arctan\left(\frac{V_{y}}{V_{x}}\right),\left|\theta_{x}-\theta_{y}\right|<90\\ -\arctan\left(\frac{V_{y}}{V_{x}}\right),\left|\theta_{x}-\theta_{y}\right|>90 \end{array}\right..$$

The deflection angle was finally sent to the external motion control unit through the serial port in real time to guide AUV docking.

## 5. Experimental Results

### 5.1. Attenuation Test of the Electromagnetic Guidance Signal Propagation 

**Figure 13:**
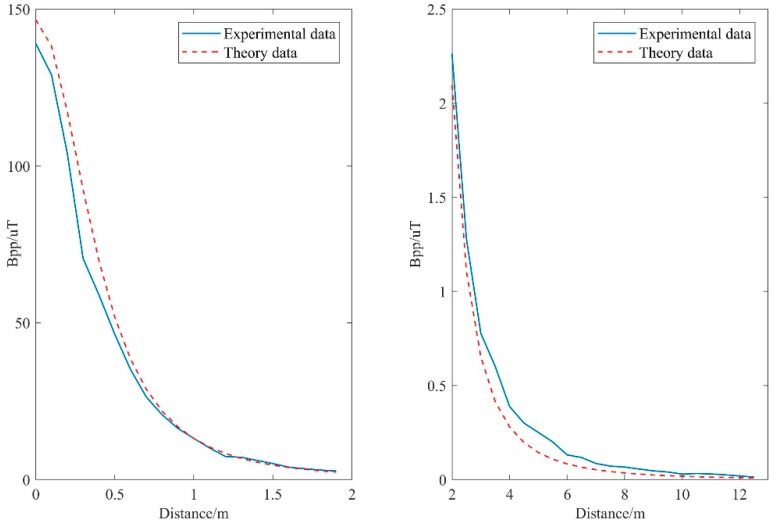
Attenuation of electromagnetic propagation in experiment and theory.

The test is mainly conducted to verify whether the magnetic field signal detected by the triaxial search coil sensor matches the theoretical value. In the test, the magnetic beacon sent a low-frequency electromagnetic signal of 1 kHz in the air and produced effective magnetic moment *M* of approximately 29.24 Am^2^. Figure 13 shows the measured peak intensity of the electromagnetic field from 0 to 12 m from the central axis and the theoretical value of the corresponding distance. The figure indicates that the measured data were basically consistent with the theoretical data, thereby verifying that the magnetic field signal obtained by the EMDG signal processing system was effective.

### 5.2. Test on Steady-State Performance

**Figure 14:**
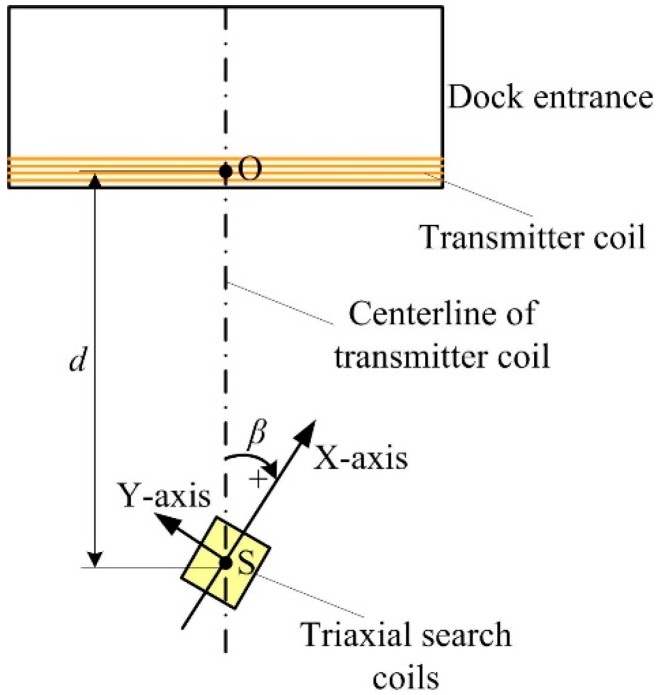
Steady-state performance test setup. The deflection angle *β* is positive in the clockwise direction. The distance from the center of the triaxial search coils S to the transmitter coil is defined as *d*.

A test was conducted to evaluate the steady-state performance of the deflection angle measurement. The experimental setup is shown in Figure 14. The center of the triaxial search coils S is placed in the centerline of the transmitter coil. The distance from S to the transmitter coil is defined as *d*. In the test, the *X*-axis and *Y*-axis of the sensor coil are placed in the horizontal plane. According to the magnetic dipole model, the magnetic fields that start from the centerline of the transmitter coil are coincidence with the centerline. Therefore, the angle between *X*-axis of the sensor coil and the centerline equals to the deflection angle *β.*

**Figure 15:**
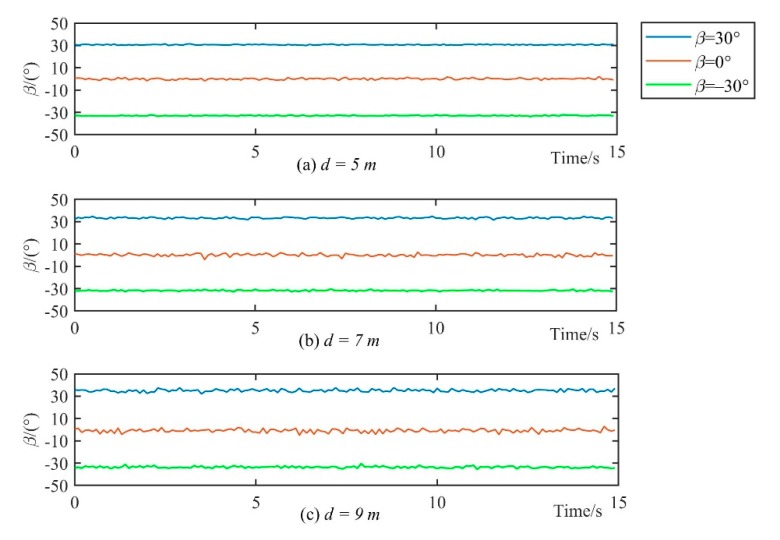
Detection of steady-state performance.

During the steady-state performance test, the search coil sensor was placed 5 m, 7 m, and 9 m away from the magnetic beacon; that is *d* = 5 m, 7 m, and 9 m, respectively. At each test point, the deflection angle *β* was set to around –30°, 0°, and 30° to get time series of the angle measurements. Figure 15 shows that the overall output of the angles at each test point had no large fluctuation. A comparison of the output performance of the angles in different distances shows that the fluctuation became large with the increase in distance. This is because when the test distance is far, the signal-to-noise ratio is small and the interference of the resolved angle is large. This phenomenon was evident at *β =* 0°, in which the *Y*-axis coil was in an almost vertical position with the magnetic field and the received signal was weak, resulting in the remarkable influence of the noise. The angle fluctuation analysis is shown in Table 1. 

When the distance *d* was 5 and 7 m, the average angle fluctuated within 1° and reached a large fluctuation range only at *d =* 9 m with *β =* 0°, which was approximately 1.23°. The steady-state performance test shows that the search coil sensor has good measurement stability.

### 5.3. Test on Deflection Angle Measurements

**Figure 16:**
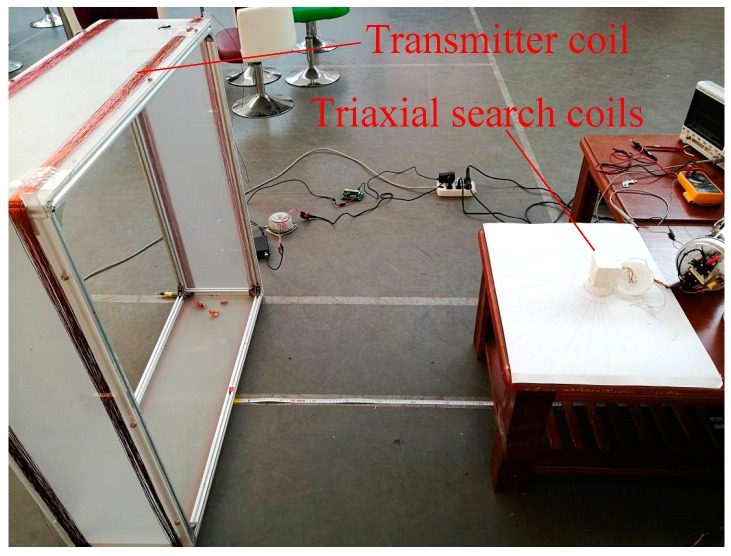
Test on deflection angle measurement.

The deflection angle *β* is the datum for docking guidance. In order to evaluate how accurately the magnetic sensor can measure the deflection angle, a test on deflection angle measurement was conducted. The experimental setup was almost the same as the test on steady-state performance. The difference was that the actual deflection angle was measured accurately by a protractor in the deflection angle measurement test. Figure 16 shows the scene of the test. In the test, we defined five test points, where *d* = 2 m, 3 m, 5 m, 7 m, and 9 m. At each test point, the deflection angles were set to –80°, –60°, –40°, –20°, 0°, 20°, 40°, 60°, 80°, respectively. Then for each preset deflection angle, approximately 10 measurements were taken. 

**Figure 17:**
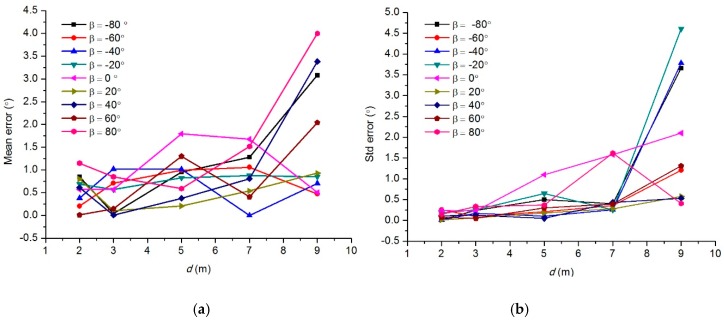
Mean angular measurement errors and error standard deviation of the test points. (**a**) Mean angular measurement errors. (**b**) Error standard deviation.

Figure 17 shows the mean angular measurement error and error standard deviation of the test points. We can see from Figure 17a that when d ≤ 7 m, the mean angular measurement error was less than 1.3°, with a standard deviation less than 0.5° in most of the cases. But the curves of *β* = 0° indicate an exception. When *β* = 0° and d ≤ 7 m, the maximum angular measurement error is 1.8° and the maximum error standard deviation is 1.6°. This is because in the case of *β* = 0°, the output in Y-coil of the magnetic sensor *V_y_* = 0 theoretically. However, due to the magnetic noise and the cross-talk, *V_y_* is nonzero, which also means that in *Y*-axis, the signal-to-noise ratio is low. 

Therefore, according to Equation (21), the nonzero V_y_ will cause an additional angular measurement error. In addition, we can see from the figure that when d > 7 m, the measurement error becomes larger. This does not affect the magnetic guidance, because when the AUV is far from the dock, a coarse deflection angle is enough. These test results indicated that the accuracy of deflection angle measurements meets the requirement of AUV docking. 

### 5.4. Docking Experimental Setup

AUV docking tests under water were performed to verify the docking ability of the EMDG system, including tests in normal docking position and extreme position where the AUV cannot “see” the dock entrance. The test site was an approximately 0.67 m deep pool in Hangzhou Dianzi University. A part of the area, which was 25 m long and 7.6 m wide, was taken as the testing zone. 

**Figure 18:**
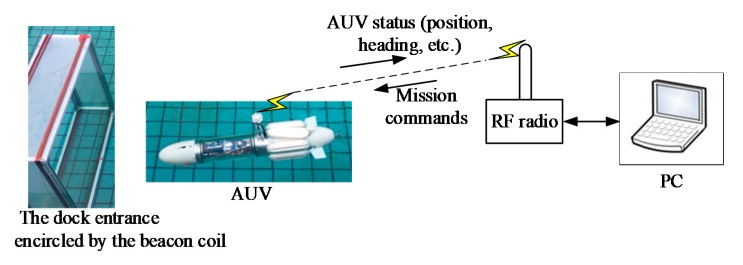
Docking experimental setup.

The docking experimental setup is shown in Figure 18 [23]. The entrance of the dock is a square with side length of 0.95 m. In the experiments, the dock entrance encircled by the beacon coil was placed in the water, whereas the other part of the magnetic beacon was placed on land. The magnetic beacon emitted a low-frequency electromagnetic signal of 1 kHz and produced an effective magnetic moment of approximately 29.24 Am^2^. The AUV moved near the water surface with only its antenna outside the water surface. A laptop PC communicated with the AUV by an RF transceiver that sends mission commands and receives AUV status updates, such as position and deflection angles. This data was recorded in PC at a frequency of 2 Hz. 

The AUV for docking test is a self-designed micro AUV [23], which has an overall length of 880 mm and a hull diameter of 130 mm. The AUV is portable with an 11 kg weight in air, and controls the yaw and pitch using control fins and steering jet pumps. The main vehicle specifications of the test bed AUV are shown in Table 2. The triaxial search coils were installed in the nose of the AUV with their three axes aligned with the principle axes of the AUV, and the signal conditioning and processing subsystem was in the electronic housing. The location of them can also be seen in Figure 1. 

During the docking experiments, part of the transmitter coil is outside the water and part of the magnetic field will go outside the water. However, this has little influence on the AUV docking. The reason is as follows: The receiver coil in the nose of the AUV was totally immersed in water and the center of the dock entrance (transmitter coil) was under the water. According to the characteristics of the magnetic dipole model, the magnetic fields that go outside of the water are the upper half of the magnetic fields. These fields tend to go upward and will not be sensed by the AUV; that is, the AUV only sensed the magnetic fields that come from the water. 

In the tests, we found that the jet pumps installed in the nose of the AUV have great electromagnetic disturbance on the magnetic sensor. This is probably because the magnetic receiver is mounted near the jet pumps. Hence, the jet pumps are used for higher maneuvering only when the deflection angle is larger than a preset angle. When the deflection angle is small, only the control fins are used for precise maneuvering. In the situation when the jet pumps are used, we used a time–division method to avoid the disturbance. When the jet pumps are working, the deflection angles sensed by the sensor are abandoned. The jet pumps then stop pumping for a while at 1-second intervals, and during the pump–stop time, the magnetic sensor measurements are used for docking guidance.

### 5.5. AUV Electromagnetic Guidance Docking Tests in Normal Position

During the docking tests in normal position, the start point of proximal docking stage is chose to be approximately 20 m ahead of the dock entrance. To illustrate the ability for docking guidance, the relative angle of the AUV to the dock centerline was set to around −45° in the start point. Then, the AUV attempted to sense the magnetic field emanating from the dock entrance, resolved the deflection angles. If the AUV detects the magnetic field, then it tries to follow the magnetic field lines into the dock. However, if the AUV does not detect the magnetic field, then it remains in its original heading.

**Figure 19:**
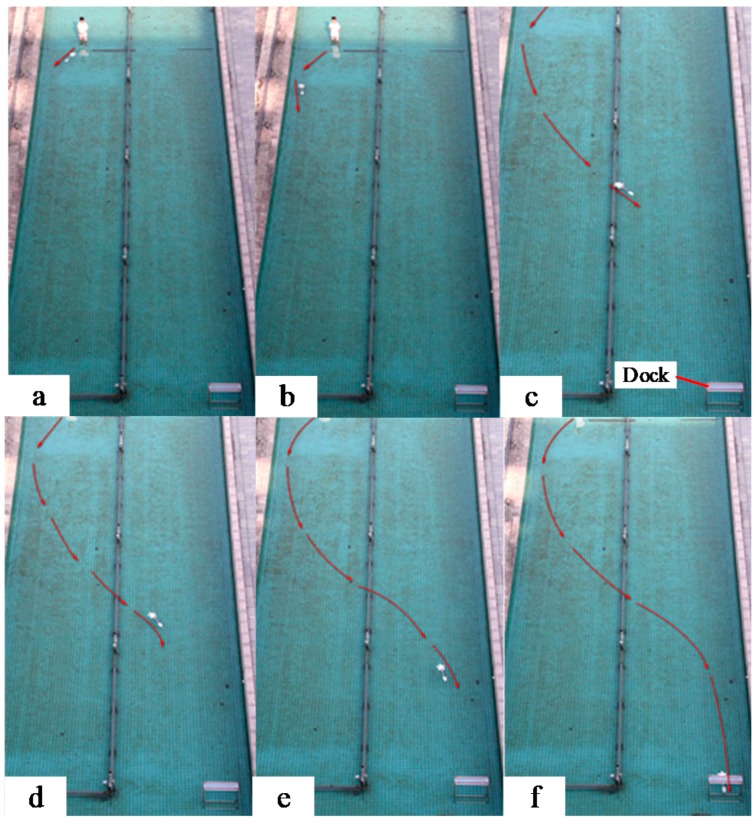
Sequence of frames from the camera showing the underwater docking process. The AUV started beyond 20 m away from the dock entrance. (**a**) Relative angle of the AUV to the dock centerline was set to around −45° at the starting point; (**b**) AUV detected the magnetic field from the beacon and then attempted to maneuver to head toward the dock; (**c**–**f**) AUV followed the magnetic field line and entered the dock.

**Figure 20:**
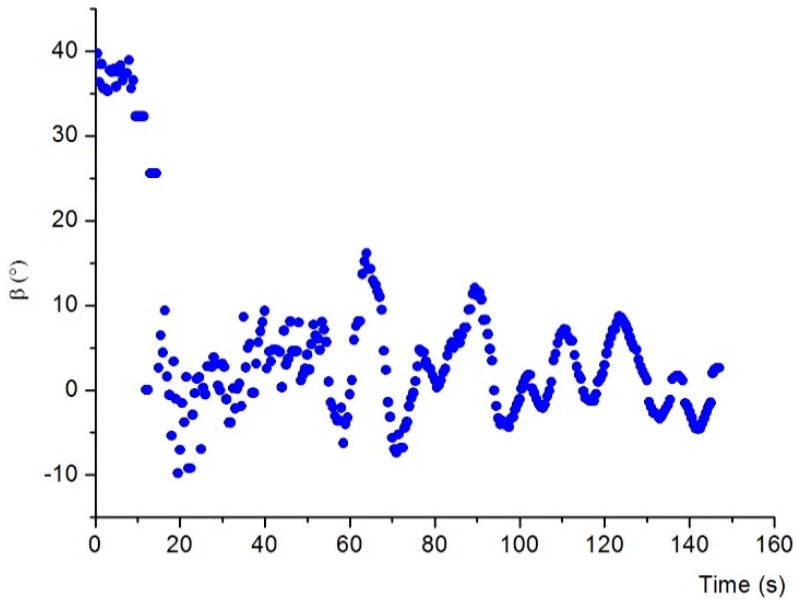
Deflection angle measured by the magnetic sensor during docking in normal position.

Figure 19 shows the sequence of frames from the camera showing one of the docking tests. In Figure 19a, the relative angle of the AUV to the dock centerline was set to around –45° at the starting point. In Figure 19b, the AUV detected the magnetic field from the beacon and then attempted to maneuver to head toward the dock; In Figure 19c–f, the AUV tried to follow the magnetic field line and entered the dock. Figure 20 shows the deflection angle measured by the magnetic sensor during docking. We can see from the figure that the deflection angle was about 40° in the start point, and then it became smaller during the docking process. Twenty docking tests were conducted in the process, with guidance being successfully performed in 17 tests. Repeated tests showed that the system achieved AUV docking guidance within 20 m, thereby meeting the requirements of proximal docking guidance.

### 5.6. AUV Docking Test in Extreme Position

**Figure 21:**
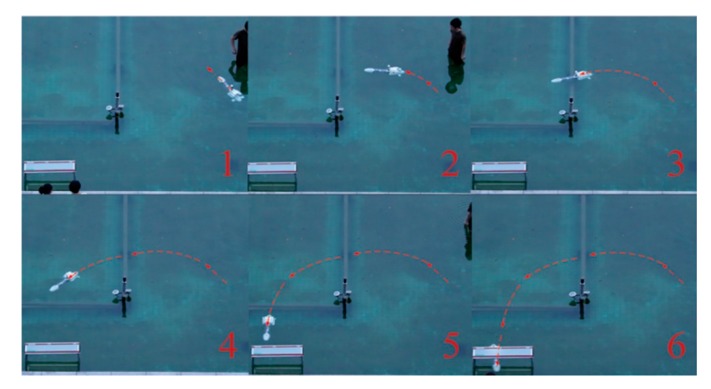
AUV docking test in extreme position.

The extreme position means that the AUV is close to magnetic beacon, which is not in the horizontal view of AUV. As shown in Figure 21, that is, the location of magnetic beacon is beyond the sight angle range of the AUV front camera or the acoustic sensor. In such an extreme position, AUV cannot evidently realize docking through normal optical or acoustic guidance. However, the electromagnetic guidance can still capture the magnetic signal in the extreme position because the magnetic field line emits by the magnetic beacon is close.

**Figure 22:**
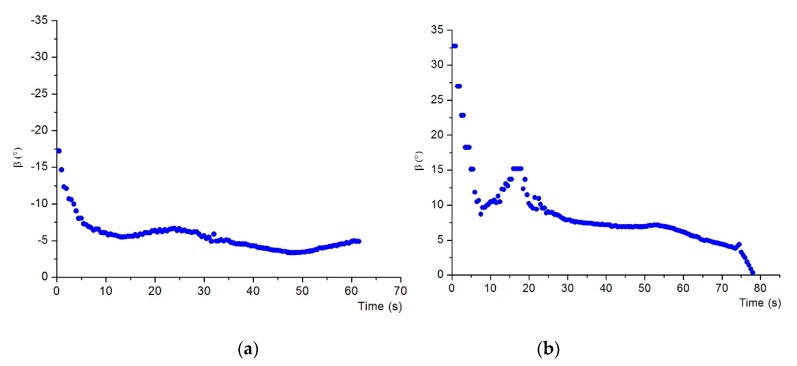
Deflection angle measured by the magnetic sensor during docking in extreme position. (**a**) Docking from left-hand side of the dock entrance. (**b**) Docking from right-hand side of the dock entrance.

Figure 21 shows the successful electromagnetic guided docking by the AUV in the extreme position. Figure 22 shows the deflection angle measured by the magnetic sensor during docking, where in Figure 22a, the AUV started from the left-hand side of the dock entrance, and in Figure 22b, the AUV started from the right-hand side of the dock entrance. We can see that the deflection angle was decreased to around zero when the docking was finished. In the docking experiment in an extreme position, the AUV needs to regulate the course in a large area and short distance, which demands high requirements on the real-time guidance and maneuverability of the AUV. This system has an output capacity of guiding information for 10 times at 1 s, which can meet the real-time guidance requirement. In addition, the auxiliary steering of steering jet pumps of the AUV can achieve course regulation in a large range with low speed. Therefore, this system has docking guidance capability in an extreme position, which cannot be realized by optical or other guidance methods. Instead of returning the AUV to the front of the docking station and restarting the docking process as the conventional practice, this situation is especially applicable for rapid docking again after AUV docking failure.

## 6. Conclusions

A compact and low-cost EM docking guidance (EMDG) system for micro AUVs was designed, built, and tested. The EMDG system is composed of a transmitter coil located on the dock and a three-axial search coil magnetometer acting as a receiver. The search coil magnetometer was optimized for small size while maintaining sufficient sensitivity. The signal conditioning and processing subsystem in the EMDG system was designed to be capable of two-level automatic gain amplification to adaptively amplify the received signal. Then, a digital orthogonal lock-in amplifier algorithm was used to calculate the magnetic signal amplitude and its phase, which were used to calculate the deflection angle *β* for docking guidance.

The underwater docking tests showed that the system can detect the electromagnetic signal and successfully guide AUV docking; thus, it meets the requirements of proximal docking guidance. Moreover, the docking test in an extreme position showed that the EMDG system can still guide the AUV into the dock, a task that cannot be realized through normal optical or acoustic guidance. 

Given the inherent advantages of EM guidance, the proposed system demonstrates robust operation under complex underwater conditions. The main contributions of this work are as follows:

(1) This study is the first to focus on the EM guidance system for low-cost micro AUVs. With a large number of low-cost micro AUVs with EM docking capability, large-scale sampling data with sufficient spatiotemporal resolution can be achieved.
(2) The proposed EMDG system has docking guidance capability in extreme positions where the optical or acoustic sensors cannot “see” the dock entrance. This situation typically occurs when an AUV fails to enter the dock entrance during the final docking stage. In this situation, an AUV with optical or acoustic guidance has to return to the docking start point, which is approximately 10–20 m ahead of the dock entrance, and restart the docking process. By contrast, the AUV with EMDG system can conduct rapid docking again after AUV docking failure.
(3) The designed search coil sensor in the AUV is inexpensive. The cost is estimated to be less than 90 dollars. About 70% of this cost is for customization, such as 3D printing. The cost will be substantially reduced if the search coil sensor undergoes volume production.
(4) The search coil sensor was optimized for small size while maintaining sufficient sensitivity. In literature [16], the search coil sensor of the EM guidance system consists of three orthogonal coils with each coil having a 90 mm diameter. The sensor was mounted in a mid-size Odyssey IIb AUV with a 580 mm diameter. Because the sensor has a large volume, it is not suitable for some low-cost portable micro AUVs, which have a typical hull diameter of (110–150) mm. By contrast, in the present study, with each sensor coil having a 25 mm diameter, the triaxial searching coil sensor has a dimension of only 56 mm × 56 mm × 56 mm. Thus, the system can be equipped on a wide range of AUVs, including low-cost micro ones.

Future studies can focus on docking guidance and control algorithms.

## Figures and Tables

**Table 1:** Fixed-angle fluctuation analysis.

Distance *d* (m)	Deflection Angle *β*		
	−30°	0°	30°
5	0.2348	0.59677	0.2127
7	0.5171	0.8903	0.3997
9	0.9331	1.2307	0.6737

**Table 2:** Main vehicle specifications of the test bed AUV [23].

Length	Approximately 880 mm
Diameter	130 mm
Weight in air	11 kg
Communications	RF transceiver, WiFi
Navigation	GPS, Pressure sensor, MEMS Inertial Measurement Unit
Vehicle control	Magnetic coupling control fins and steering jet pumps
Battery	Lithium Battery, 20.4 Ah
